# Does vitamin blends supplementation affect the animal performance, carcass traits, and nutrient digestibility of young Nellore finishing bulls?

**DOI:** 10.5713/ab.23.0087

**Published:** 2023-08-23

**Authors:** Dhones Rodrigues de Andrade, Flávia Adriane de Sales Silva, Jardeson de Souza Pinheiro, Júlia Travassos da Silva, Nathália Veloso Trópia, Leticia Artuzo Godoi, Rizielly Saraiva Reis Vilela, Fernando Alerrandro Andrade Cidrini, Luciana Navajas Rennó, Diego Zanetti, Tiago Sabella Acedo, Sebastião de Campos Valadares Filho

**Affiliations:** 1 Department of Animal Science, Universidade Federal de Viçosa, Campus Viçosa, Minas Gerais, 36570-000, Brazil; 2 Department of Animal Science, Instituto Federal de Educação, Ciência e Tecnologia do Sul de Minas Gerais, Campus Machado, Minas Gerais, 37750-000, Brazil; 3 DSM Nutritional Products Brazil S.A., São Paulo, SP 01541-905, Brazil

**Keywords:** B Complex, Cattle, Tocopherol, Vitamin Supplementation, Zebu

## Abstract

**Objective:**

This study was realized to evaluate the effects of supplementation with blends of water and fat-soluble vitamins on animal performance and carcass traits of young Nellore bulls.

**Methods:**

Forty-three Nellore bulls, with an initial weight of 261±27.3 kg and a mean age of 8±1.0 months, were used. Five animals were slaughtered at the beginning of the experiment (reference group), to determine the initial empty body weight of the bulls that remained in the experiment. The remaining 38 bulls were fed *ad libitum* and distributed in a completely randomized design in a 2×2 factorial scheme, with or without supplementation of water-soluble (B-blend+ or B-blend−) and fat-soluble (ADE+ or ADE−) vitamin blends. Diets were isonitrogenous (120 g of crude protein/kg dry matter [DM] of total mixed ration) and consisted of a roughage:concentrate rate of 30:70 based on total DM of diet. The experiment lasted 170 days, with 30 days of adaptation and 140 days for data collection. At the beginning and end of the experimental period, the bulls were weighed to determine the average daily gain. To estimate the apparent digestibility of nutrients and microbial efficiency, spot collections of feces and urine were performed for five consecutive days.

**Results:**

DM, ashes, organic matter, crude protein, ethereal extract, neutral detergent fiber corrected for residual ash and residual nitrogenous, and N intake and apparent digestibility were not influenced by vitamin supplementation, but total digestible nutrients intake and non-fibrous carbohydrates digestibility were influenced by B complex vitamin supplementation. Nitrogen balance, microbial efficiency, and performance data were not influenced (p>0.05) by vitamin supplementation.

**Conclusion:**

Vitamin supplementation (a blend of water-soluble and fat-soluble vitamins or their combinations) does not influence the animal performance and carcass traits of young Nellore bulls.

## INTRODUCTION

Vitamins are essential metabolic catalysts used in animal diets [1], being fundamental in meeting the physiological demands related to the immune processes (health), growth, and reproduction [2]. In beef cattle, the vitamins commonly supplemented include the vitamins A, D, and E [3], and water-soluble vitamins, specifically B1 (thiamine), B3 (niacin), and B7 (biotin) [4].

Briefly, vitamin A is involved in the formation, regeneration, and protection of the ectoderm and mucous membranes. Vitamin E promotes improvement in antibody formation and humoral resistance; it is necessary for cell metabolism and acts as an antioxidant of unsaturated fatty acids and vitamin A on meat quality. Vitamin D, regulates calcium (Ca) and phosphorus (P) homeostasis, increasing intestinal uptake and bone reabsorption, favoring the increase of Ca-dependent proteolytic enzymes, which can lead to an improvement in meat quality [3].

Lastly, water-soluble vitamins, particularly thiamine (B1), niacin (B3), and biotin (B7), play important roles as coenzymes in the metabolism of carbohydrates, proteins, and lipids, acting in the process of hepatic detoxification of ammonia into urea, as well as in the metabolism of ketone liver; increase protein synthesis by ruminal microorganisms and enzymatic carboxylation, which is responsible for providing energy to the body [5].

The literature reports varied effects of vitamin supplementation for finishing cattle on animal performance and carcass traits [6–8]. For example, according to Bryant et al [9] and Baldin et al [10], the supplementation of fat-soluble vitamins alone (A) or associated (D and E) did not influence animal performance and carcass traits of finishing bulls. On the other hand, some studies have demonstrated better productive performance of bulls [11] and buffaloes [12] in response to water-soluble vitamin supplementation (vitamins B1 and B3).

The authors performed a search of articles that used vitamin supplementation for beef cattle in the last 10 years, evaluating the effects of this additive on performance and carcass traits. Only 7 articles used some of the vitamins used in the present study. Of the 7 articles found, only 14% found positive effects (better carcass yield) and 86% had no effects. In addition, no study was found that evaluated the effect of water-soluble vitamins in isolation or through a blend, with the objective of evaluating performance and carcass traits.

Thus, we hypothesized that supplementation with blends of vitamins A, D, and E, and vitamins of the B complex (B1, B3, and B7) or their combinations improves the animal performance and carcass traits of young Nellore bulls. The objective this study was to evaluate the effects of supplementation of a B vitamin blend (biotin, niacin, and thiamine), fat-soluble vitamin blend (ADE), or a combination with these two blends on animal performance, nitrogen balance, microbial efficiency, nutrient digestibility and carcass traits of young bulls Nellore finished in feedlot.

## MATERIALS AND METHODS

### Animal care

The Ethics Committee on the Use of Production Animals of the Universidade Federal de Viçosa approved all procedures involving animals (protocol N° 037/2018).

### Animal handling, experimental design, and diets

The experiment was conducted at the Experimental Feedlot of the Department of Animal Science at the Universidade Federal de Viçosa (UFV), Viçosa, Minas Gerais, Brazil.

Forty-three Nellore bulls with an average age of 8±1.0 months and an average body weight (BW) of 261±27.3 kg were utilized. The experiment was conducted as completely randomized design in a 2×2 factorial scheme. The factors consisted of two fat-soluble vitamins blend (ADE) supplementation levels (ADE− or ADE+) and two B vitamin blend (biotin, niacin, and thiamine) supplementation levels (B-blend− or B-blend+). Thus, the treatments evaluated consisted of no vitamin supplementation (ADE− B-blend−), supplementation of a B vitamin blend (ADE− B-blend+), supplementation with a fat-soluble vitamin blend (ADE+ B-blend−), or supplementation with a combination of these two blends (ADE+ B-blend+). The experiment lasted 170 days with 30 days for the bulls to adapt to the location and diets and 140 days for data collection.

Bulls were identified, weighed, treated against endo and ectoparasites, and housed in 50 m^2^ collective pens with concrete floors, which were equipped with electronic feeders and drinkers (model AF-1000 Master; Intergado Ltda., Contagem, Minas Gerais, Brazil [13]. Aiming to avoid ruminal diseases, the animals were adapted during 30-d. Thus, the adaptation of the bulls to the final ratio of roughage:concentrate was carried out as follow: bulls received the diet with 20% of the total meal concentrate during the first five days, 30% concentrate between 6th to 10th d of the adaptation period, the diet with 40% concentrate between 11th – 15th d of the adaptation period, 50% of concentrate between 16th – 20th d of the adaptation period, 60% of concentrate between 21th – 25th d of the adaptation period and lastly 70% of concentrate between 26th – 30th d of the adaptation period, totaling 30 days of adaptation. At the end of the adaptation period, the bulls were weighed after a 16-h solid fasting period and were randomized into two groups: baseline (5 bulls) and experimental (38 bulls). The five bulls in the baseline group (BW = 261±33.7 kg) were slaughtered at the beginning of the experiment to measure empty BW (EBW) and to estimate the initial EBW of the remaining experimental bulls. The 38 bulls designed to vitamin supplementation evaluation were randomly distributed into four groups, being two groups with 10 bulls and two groups with 9 bulls each, that were randomly assigned to one of four vitamin supplementation strategies.

The roughage:concentrate ratio was 30:70 (dry matter [DM] basis; Table 1) and diets were formulated to meet animals’ requirements according to the BR-CORTE system [14], targeting an average daily gain (ADG) of 1.2 kg/d (Table 2). Vitamin levels were according to vitamin supplementation guidelines [15] proposed by DSM Nutritional Products Ltd (Basel, Switzerland). Vitamin blends were premixed into the concentrate, and the levels of each vitamin per kg of diet DM were: 3.3 mg of biotin (D-biotin), 111.1 mg of niacin (niacin), 28.9 mg of thiamine (thiamine hydrochloride), 6,666.7 IU of vitamin A (retinyl acetate), 5,111.1 IU of vitamin D (13% D3-cholecalciferol and 87% 25-Hydroxyvitamin D3 - Hy-D), and 70 IU of vitamin E (DL alfa-tocopheryl acetate). The experimental mineral supplements were produced at a commercial feed meal plant following all the manufacturing standards (DSM Nutritional Products Brazil S.A., Mairinque, SP, Brazil). All vitamins used in the study were also from DSM Nutritional Products Brazil S.A.

Corn silage and concentrate were weighed separately, then mechanically mixed at the time of feeding such a total mixed ration was provided ad libitum twice a day (07:00 and 16:00 hours) and the bulls had free access to water. The diet was adjusted daily to allow approximately 5% refusal of the offered total on an as-fed basis. Refusals were mixed with the new feed that was offered the next day.

The bulls were weighed every 28 days to monitor the ADG. For quantification of their performance, the first and last weights (days 1 and 140, respectively) were used, which were preceded by a 14-h solid fasting period.

### Collections, digestibility assays, and slaughter procedures

Corn silage was sampled daily and stored at −20°C until further analysis. Forage samples were combined weekly (percent as-fed basis), dried in a forced-air oven (55°C) for 72 h, and ground through a 2- and 1-mm screen (Fortinox, Piracicaba, São Paulo, Brazil). Next, the total DM of these samples was evaluated according to AOAC [16] by using the method 934.01. Based on the DM content of corn silage, composite samples were assembled proportionally to the amount of offered roughage during 4 weeks. The individual ingredients used to make the concentrate were sampled directly from the feed mill silos on the days the concentrate was mixed. These ingredients were analyzed individually and used to calculate the diet composition.

To evaluate the total apparent digestibility of nutrients, N balance, and microbial efficiency, spot fecal and urine samples were collected from all bulls on d 16 to 20, d 72 to 76, and d 129 to 133. Fecal samples were collected during five consecutive days at 06:00 on d 1, at 09:00 on d 2, at 12:00 on d 3, at 15:00 on d 4, and at 18:00 on d 5. Urine samples were collected at 12:00 on d 3 and at 18:00 on d 5. Fecal samples obtained directly from the rectum of the animals. Approximately 250 g of feces were collected per sampling time and oven-dried (55°C for 72 h). Subsequently, a composite sample was performed for each animal in each collection period. The indigestible neutral detergent fiber (iNDF) was used as a marker to estimate the fecal DM production and then calculate the apparent digestibility of nutrients.

The urine was collected using collection cups. At the end of each collection, two urine samples were taken. We obtained a 10-mL sample that was diluted with 40 mL of 0.036 N H_2_SO_4_ to avoid losses of allantoin and uric acid. Another 20-mL sample was collected without dilution to quantify the total N content. Subsequently, for both cases, a composite was performed per animal and collection period. Urine composite samples were stored at −20°C until further analysis.

At the end of the experiment, all bulls were slaughtered at the UFV after a 16-h solid fasting period. The slaughter was performed via stunning and severing of the jugular vein for total bleeding. The digestive tract of each bull was emptied and washed, and each organ was weighed separately. The weight of the non-carcass components (NCC) was composed of the sum of the weights of the heart, lungs, liver, spleen, kidneys, internal fat, diaphragm, mesentery, tail, tongue, trachea, esophagus, reproductive tract, washed gastrointestinal tract, head (no leather), leather (body, head, and limbs), hooves and blood. The NCC was added to the carcass weight to determine the EBW. The carcass of each bull was divided into two halves that were weighed (hot carcass weight; HCW) to evaluate the hot carcass yield (HCY) and cooled at 4°C for 24 h. After this time, carcasses were weighed (cold carcass weight; CCW) to evaluate the cold carcass yield (CCY). The carcass length (CL) was measured as the distance from the cranial edge of the ischiopubic symphysis to the medial cranial edge of the first rib. The *Longissimus lumborum* muscle area (LMA) and 12th-rib fat were measured between the 12th and 13th ribs on the left half-carcass. To determine body chemical composition, samples from the NCC and the section between the 9th and 11th rib called as HH section [17], of the left carcass of each bull were collected, weighed, dissected and lyophilized ([16]; method 934.01). After the samples were processed, chemical analyzes of the HH section and NCC were carried out.

### Laboratory analyses and calculations

The samples of corn silage, concentrate ingredients, and feces were analyzed for their contents of DM ([16]; method 934.01), organic matter (OM; [16]; method 930.05), total N ([16]; method 981.10), ethereal extract (EE; [18]; method 945.16), ashes (MM; [19]; method 924.05) and neutral detergent fiber (NDF; [20]). The NDF quantification was performed without the addition of sodium sulfite but with the addition of thermostable alpha-amylase to neutral detergent (Ankom Tech. Corp., Fairport, NY, USA). The NDF concentrations were corrected for ash [20] and residual N compounds [21]. We calculated crude protein (CP) content as the product of total N content and the factor 6.25. The iNDF content was determined according to Valente et al [22]. Non-fibrous carbohydrates (NFC) were calculated as proposed by Detmann and Valadares Filho [23] from the equation: NFC = 100–[(% CP diet–% CP derived from urea+% urea)+% NDF+% EE+% MM], where NFC = non-fibrous carbohydrates; % CP = crude dietary protein; NDF = dietary neutral detergent fiber; EE = dietary ether extract content; MM = ash content of the diet. The contents of total digestible nutrients (TDN) were estimated through the sum of the digestible nutrients, where: TDN = digestible CP+2.25× digestible EE+digestible NDF+digestible NFC [24].

Urine samples were analyzed for creatinine, uric acid, and allantoin. The analyses of uric acid and creatinine were performed by using an automatic biochemical analyzer (Minudray/model BS200E, Shenzhen, China), whereas the allantoin analysis was performed according to the colorimetric method described by Chen and Gomes [25].

The daily excretion of creatinine (EC; g/d) was calculated based on the following equation (EC (g/d) = 0.0345×BW^0.9491^ ×1,000, where BW = body weight; [26]). Then, the daily urinary volume (L/d) was estimated from the ratio between the estimated EC (g/d) and creatinine concentrations in the spot urine sample (g/L).

Total excretion of purine derivatives was calculated as the sum of the amounts of allantoin, and uric acid excreted in the urine, which was obtained by the product of their concentration in the urine and the urinary volume. Absorbed purines and ruminal synthesis of nitrogen compounds were calculated according to Barbosa et al [27].

The microbial crude protein (MCP; g/d) was calculated by the product of the ruminal synthesis of nitrogen compounds and factor 6.25. The microbial efficiency was calculated by the ratio between the MCP and TDN and digestible OM (DOM) intakes, expressed in g MCP/kg TDN and g MCP/kg DOM.

The N balance of the animals were also calculated. For this purpose, we calculated the intake and excretion fecal of N (g/d) as the ratio of N content in the diets (by DM bases) and feces excretion in DM bases (g/d), respectively. In addition, we calculated N urinary excretion (g/d) as the difference N consumed less N fecal and, N retained as described by Cole et al [28], which we obtained through comparative slaughter, whose procedures we described in the slaughter procedures.

### Statistical analysis

Data were analyzed in a completely randomized design using a 2×2 factorial scheme by PROC MIXED of SAS (version 9.4):


$${\text{Y}}_{\text{ijk}}=\mu +{\text{F}}_{\text{i}}+{\text{B}}_{\text{j}}+\text{F}\times {\text{B}}_{\text{k}}+{\text{e}}_{\text{ijk}}$$

where: **μ** = general constant; **F_i_** = fixed effect of fat-soluble vitamin blend (ADE; supplementation or not); **B_j_** = fixed effect of B vitamin blend (biotin, niacin, and thiamine; supplementation or not); **F×B_k_** = effect of the interaction between fat-soluble vitamin blend and B vitamin blend; supplementation or not; and **e_ijk_** = residual error associated with each observation. The treatments and interaction were added as fixed parameters in the model. Means were compared using the Student’s t-test. Non-significant interactions were omitted, and effects were considered significant when p<0.05.

The criterion adopted for identifying outliers was based on the normal distribution curve, in which values of Student's standardized residuals greater than |3| were considered as outliers and, therefore, removed from the respective database. Based on that, two bulls were removed from the analyzes (one from the ADE-/B-blend-treatment and the other from the ADE+/B-blend-treatment).

## RESULTS

### Intake, apparent digestibility of nutrients, and microbial efficiency

There was no ADE×B-blend supplementation interaction (p>0.05) for DM and nutrient intake, digestibility of nutrients and microbial efficiency. Intakes and apparent digestibility of DM, MM, OM, CP, EE, apNDF, N, and NFC were not influenced (p>0.05) by vitamin supplementation (Table 3). However, bulls fed diets containing a B vitamins blend showed lower values (p<0.05) for TDN intake (p = 0.022) and apparent digestibility of NFC (p = 0.046) compared to bulls fed diets without B vitamin blend supplementation (Table 3).

The was no interaction (p>0.05) in microbial efficiency (TDN/DOM) between ADE×B-blend supplementation. The production of microbial crude protein (MCP) and the microbial efficiency, both expressed in relation to TDN, and DOM were not influenced (p>0.05) by supplementation with different vitamin blends (Table 3).

### Nitrogen balance, animal performance, and carcass traits

There was no ADE×B-blend supplementation interaction (p>0.05) for nitrogen balance. The intake, excretion, absorption, and retention of N (g/d), as well as the ratio between retained N and N intake, were not influenced (p>0.05) by supplementation with different vitamin blends (Table 4).

There was no effect (p>0.05) in animal performance and carcass traits between ADE×B-blend supplementation. There was no effect (p>0.05) of the supplementation of different vitamin blends on the initial SBW (SBWi), final SBW (SBWf), initial EBW (EBWi), final EBW (EBWf), the weight of non-carcass components (NCW), ADG, empty body weight gain (EBWG), hot carcass gain (HCG), CCW, HCW, subcutaneous fat thickness (SFT), CL, LMA, and HCY (Table 5).

## DISCUSSION

### Intake and digestibility of nutrients and microbial production

Dry matter intake (DMI) and nutrient digestibility are the main factors that affect animal performance. The DMI accounts for 70% of the variation in digestible energy intake between animals and diets, and differences in digestibility account for the other 30% of this variation [29]. In the present study, no effects of different vitamin blends supplemented on DMI, and nutrients were observed, except for TDN intake. Vieira et al [30] did not find differences for DMI and nutrients in cattle submitted to ADE vitamin supplementation (5 mL), via injection. Bao et al [31] supplementing with vitamin E, above the level used in the present work (100 and 200 IU/kg DM) in the diets of female Sika deer, did not observe effects of vitamin E supplementation on nutrient intake and digestibility. Kandathil and Bandla [32] evaluated the oral supplementation of fat-soluble vitamins and complex B in doses: 3.66 mg/kg DMI of thiamine; 6.78 mg/kg DMI riboflavin; 16.3 mg/kg DMI niacin; 41.1 mg/kg DMI pantothenic acid; 3.87 mg/kg DMI pyridoxine; 0.323 mg/kg DMI biotin; 4.12 mg/kg DMI folic acid; 0.055 mg/kg DMI of B12 and 500 IU/kg DMI of vitamin K for Deoni cows. The authors found no differences in DMI (kg/d) between the supplemented and control groups.

We believe that the lack of effect of vitamin supplementation on most of these constituents may be associated with the constant values of the roughage:concentrate ratio (R:C) and the amount of NDF in the diets throughout the feedlot period. In general, these two factors are the ones that most influence the amount of feed ingested by animals Lima et al [29]. However, the TDN intake was lower for the animals receiving vitamin supplementation from the B-blend+ group. It is worth noting that there was a lower digestibility of NFC, influencing the lower TDN intake. This occurs because TDN is a variable calculated according to the digestibility of nutrients such as CP, EE, NDF, and NFC, where in our study the value of NFC represented 60% of the chemical composition of the diet.

Nutrient digestibility was not influenced by supplementation with different vitamin blends, except for NFC digestibility, which was affected by B complex vitamin supplementation (B-blend+). However, Luo et al [11] when supplementing Jinjiang bulls, finishing with four levels of niacin (B3) 0, 320, 480, and 640 mg niacin/kg DM, found a significant increase in the apparent digestibility of diet nutrients for the treatment 640 mg niacin/kg DM. However, the supplemental vitamin B3 values used in that study were 576% higher than those adopted in our work. In another study carried out by Kandathil and Bandla [32] using oral supplementation of liposoluble vitamins and complex B in Deoni cows at doses: 3.66 mg/kg DMI of thiamine; 6.78 mg/kg DMI riboflavin; 16.3 mg/kg DMI niacin; 41.1 mg/kg DMI pantothenic acid; 3.87 mg/kg DMI pyridoxine; 0.323 mg/kg DMI biotin; 4.12 mg/kg DMI folic acid; 0.055 mg/kg DMI of B12 and 500 IU/kg DMI of vitamin K found similar results to the present study for nutrient digestibility (CP, OM, EE, ash, and NDF), where supplementation did not affect these parameters. Although inconsistencies in the results can be seen in the literature this can be related to the composition of the diet, level, type of vitamin supplementation, experimental conditions, animal category, and clinical and nutritional status, in addition other factors.

It is well known that vitamins act as a growth factor for ruminal microorganisms [33]. In addition, there are reports in the literature that supplementation with B vitamins promotes better rumen fermentation and improves microbial protein synthesis [34,35,11]. Based on the literature, we expected some effect on MCP and microbial efficiency of bulls supplemented with B-blend(+) group vitamins. However, no effect could be noted on MCP and microbial efficiency, both expressed in relation to TDN intake and OM intake digestible. In fact, some alteration in the microbial populations must have occurred, due to the influence of the B vitamins, but possibly this does not affect the final balance of MCP.

According to Schwab et al [36], vitamin B3 supplementation may be beneficial when B3 synthesis is limited, or microbial growth is not maximal. This is directly related to some challenge that the animal is submitted to, and which ends up affecting the normal functioning of the rumen and limits the production of other vitamins by ruminal microorganisms, especially vitamins B1 and B7. Furthermore, studies in this regard have shown that the main cellulolytic ruminal microorganisms, including bacteria (Ruminococcus and Bacteroides species) and anaerobic fungi (Neocallimastix), have specific requirements for thiamine, riboflavin, niacin, pyridoxine, biotin, folic acid, and B12 [32].

In some cases, feedlot diets can result in a great challenge for the bulls, especially during the initial period of feedlot and when high levels of soluble carbohydrates are used. The use of diets with high levels of soluble carbohydrates without an adaptation protocol can modify the ruminal environment and contribute to the decline of microbial synthesis [37]. However, Luo et al [11] verified an improvement in the synthesis of microbial crude protein in the rumen, when there was a supplementation of 640 mg/kg DM of vitamin B3, which reflected in a higher ADG in the initial period of feedlot for supplemented cattle. These authors attributed this response to an improvement in microbial protein synthesis in the rumen, which resulted in an increased pool of microbial protein for the duodenum.

In the case of our study, all bulls are contemporaneous and come from the same rearing system, where they received concentrated supplementation during the rearing phase from 100 days of age (Creep-feeding). Thus, the bulls already had a ruminal microbiota adapted to receive higher concentrations of soluble carbohydrates in the diet before entering feedlot finishing. Thus, the possible stressful challenges of finishing in feedlot were attenuated. In addition, the feedlot adaptation protocol used may also have influenced the lack of response to the treatments.

### Nitrogen (N) balance, animal performance, and carcass traits

An important indicator of animal protein metabolism is N balance, in addition to being a good parameter for evaluating feeds and diets [38]. When the amount of N offered is sufficient to compensate for excretions, the N balance becomes positive. However, if the total N excretion is greater than the amount of N offered, the N balance becomes negative [38].

Intake, excretion via urine and feces, absorption and retention of N, as well the ratio between N retained and N consumed were not influenced by supplementation with different vitamin blends. However, it is important to emphasize that bulls of all treatments presented a positive N balance. Therefore, vitamin supplementation did not have a determining beneficial or harmful effect on the N metabolism of the bulls.

There are no reports in the literature on the effects of using blends of water- and fat-soluble vitamins, as well their association, on the animal performance and carcass traits of beef cattle. In the present study, the variables BWi, BWf, EBWi, EBWf, NCW, ADG, EBWG, WCG, CCW, HCW, HCY, SFT, CL, and LMA were not influenced by supplementation with different vitamin blends. Thus, vitamin supplementation above the levels recommended by NASEM [5] did not result in benefits for animal performance or carcass traits of young Nellore bulls.

Similar results were observed in previous studies using vitamins alone or together, evaluating the joint effects of vitamin D and E supplementation on animal performance and carcass traits of Nellore and Canchim bulls finished in feedlot. In this work, the bulls received daily doses of 1,300 IU/vitamin E (α-tocopherol acetate) for 67 days and 7.5×10^6^ IU/vitamin D (D3) 10 days before slaughter. The authors observed no effect of vitamin supplementation on animal performance and carcass traits. These authors suggested that there is no need for supplementation of vitamins D and E, regardless of breed, in feedlot cattle.

Gorocica-Buenfil et al [39] found no differences in the animal performance of crossbred Angus males receiving or not high vitamin A supplementation (3,500 IU/kg DM). Bryant et al [9] suggested that vitamin A supplementation for finishing steers should be carefully evaluated. According to the authors, typical feedlot diets (high grain) have an average of 5,215 IU/kg DM of vitamin A. According to the results obtained by these authors and corroborated by NASEM [5], the vitamin A requirement for finishing cattle fed similar basal diets is ≤2,205 IU/kg DM and high-concentrate diets should already meet this demand.

Wellmann et al [40] found no differences in the LMA and SFT of steers Nellore bulls submitted to doses of up to 5 times the value of vitamin A requirement recommended by NASEM [5] of 2,200 IU/kg DM, after a phase of vitamin A depletion for 91 days, to eliminate any interference of vitamin A stored in the animals. In the present study, vitamin A supplementation (6,666.7 IU/kg DM), considering the average intake of DM (6.29 kg) was 3 times higher to the NASEM [5] recommendations, and thus as the study by Wellmann et al [40] who tested up to 5 times the requirement proposed by NASEM, there was no improvement in the productive performance of the animals.

Luo et al [11], in a study evaluating supplementation with niacin (B3) for Jinjiang bulls in finishing, with levels of 0, 320, 480, and 640 mg of niacin/kg DM during a period of 56 days, found no effect on BW and DM intake. However, the average daily gain was influenced by the supplementation of 640 mg of niacin during the initial period of the experiment (1 to 28 d), which resulted in an increase in ADG of 43.75% of the animals fed with 640 mg of niacin/kg DM compared to the control group. Treatments 480 and 320 mg of niacin/kg DM were also numerically superior to the control in relation to ADG, 39.58% and 8.33%, respectively.

Supplementation with different blends of vitamins did not influence animal performance and carcass traits of the animals in the present study. The results have demonstrated that the different vitamin blends did not affect performance variables in young Nellore bulls. The vitamin requirements of growing animals are low in relation to macronutrients [5], although meeting them is very important. Thus, the concentrations of vitamins and pro-vitamins in the diet must have been sufficient to meet the requirements of growing animals and offering higher concentrations of vitamins does not promote gains in the animals' performance variables. Furthermore, the interactive effect of vitamins does not seem to have any effect on productive performance either.

## CONCLUSION

In general, vitamin supplementation (blend of water-soluble and fat-soluble vitamins or their combinations) does not influence the animal performance and carcass traits of young Nellore finishing bulls.

## Figures and Tables

**Table 1:** Chemical composition of ingredients used in experimental diets

Ingredients	DM	OM	CP	EE	apNDF	NFC	NDFi

		(g/kg DM)					
Corn silage	302.26	942.21	61.23	21.33	480.06	379.60	150.04
Ground corn	881.95	987.42	84.12	35.30	114.64	753.35	21.26
Soybean meal	882.56	931.84	502.19	6.56	136.79	286.30	16.02
Urea^1)^	994.07	994.10	2657.96	0.00	0.00	0.00	0.00
Mineral premix^2),3)^	986.24	107.06	0.00	0.00	0.00	0.00	0.00
Virginiamicin^4)^	996.18	21.90	0.00	0.00	0.00	0.00	0.00

DM, dry matter; OM, organic matter; CP, crude protein; EE, ether extract; apNDF, neutral detergent fiber corrected for residual ash and residual nitrogenous; NFC, non-fiber carbohydrates; NDFi, indigestible neutral detergent fiber.

1) Urea/ammonium sulfate ratio was 9:1.

2) Basic composition of the product: calcium carbonate, sulfur carbo-amino-phosphochelate, potassium chloride, sodium chloride (common salt), ventilated sulfur (flower of sulfur), dicalcium phosphate, magnesium oxide, antioxidant additive (BHT), biotin, cobalt carbo-amino-phosphochelate, copper carbo-amino-phosphochelate, chromium carbo-amino-phosphochelate, manganese carbo-amino-phosphochelate, selenium carbo-amino-phosphochelate, zinc carbo-amino-phosphochelate, calcium iodate, sodium monensin, vitamin A, vitamin D3, vitamin E, vitamin B1, niacin.

3) Guarantee levels per kg of the product: calcium (min), 160.00 g/kg; calcium (max), 185.00 g/kg; phosphorus (min), 20.80 g/kg; sulfur (min), 31.25 g/kg; magnesium (min), 20.80 g/kg; potassium (min), 31.25 g/kg; sodium (min), 68.75 g/kg; cobalt (min), 10.40 mg/kg; copper (min), 679.00 mg/kg; Chromium (min), 8.35 mg/kg; iodine (min), 34.50 mg/kg; manganese (min), 1,333.00 mg/kg; selenium (min), 8.35 mg/kg; Zindo (min), 2,500.00 mg/kg; Vitamin A (min), 500,000.00 IU/kg; vitamin D3 (min), 383,500.00 IU/kg; vitamin E (min), 5,250.00 IU/kg; Vitamin B1 (min), 2,165.00 IU/kg; biotin (min), 250.00 mg/kg; niacin (min), 8,300.00 IU/kg; monensin sodium, 1,733.00 mg/kg; fluoride (max), 208.00 mg/kg.

4) Virginiamicin V-max 2 (2%).

**Table 2:** Concentrate and diet ingredient proportions and chemical composition of concentrates and diets on a dry matter basis

Item	Concentrate^1)^				Diets^1)^			
	
	ADE^(−)^		ADE^(+)^		ADE^(−)^		ADE^(+)^	
			
	B-blend^(−)^	B-blend^(+)^	B-blend^(−)^	B-blend^(+)^	B-blend^(−)^	B-blend^(+)^	B-blend^(−)^	B-blend^(+)^
	Proportion of ingredients (g/kg in DM)							
Corn silage	-	-	-	-	300.4	300.4	300.4	300.4
Ground corn	906.1	906.1	906.1	906.1	634.0	634.0	634.0	634.0
Soybean meal	55.0	55.0	55.0	55.0	38.5	38.5	38.5	38.5
Urea	14.2	14.2	14.2	14.2	9.9	9.9	9.9	9.9
Mineral premix	22.9	22.9	22.9	22.9	16.0	16.0	16.0	16.0
Virginiamicin	1.8	1.8	1.8	1.8	1.2	1.2	1.2	1.2
	Chemical composition (g/kg in DM)							
DM	885.9	885.9	885.9	885.9	560.2	560.2	560.2	560.2
Ashs	37.3	37.3	37.3	37.3	43.4	43.4	43.4	43.4
OM	962.7	962.7	962.7	962.7	956.6	956.6	956.6	956.6
CP	142.6	142.6	142.6	142.6	118.2	118.2	118.2	118.2
EE	32.5	32.5	32.5	32.5	29.1	29.1	29.1	29.1
apNDF	113.4	113.4	113.4	113.4	223.4	223.4	223.4	223.4
NFC	686.7	686.7	686.7	686.7	594.6	594.6	594.6	594.6
N	22.8	22.8	22.8	22.8	18.7	18.7	18.7	18.7
	Supplementary vitamins (Premix OVN)							
Vit A (UI/kg DM)	0	0	9,523.86	9,523.86	0	0	6,666.70	6,666.70
Vit D (UI/kg DM)	0	0	7,301.57	7,301.57	0	0	5,111.10	5,111.10
Vit E (UI/kg DM)	0	0	100.00	100.00	0	0	70.00	70.00
B1 (mg/kg DM)	0	41.29	0	41.29	0	28.90	0	28.90
B3 (mg/kg DM)	0	158.71	0	158.71	0	111.10	0	111.10
B7 (mg/kg DM)	0	4.71	0	4.71	0	3.30	0	3.30

DM, dry matter; OM, organic matter; CP, crude protein; EE, ether extract; apNDF, neutral detergent fiber corrected for residual ash and residual nitrogenous; NFC, non-fiber carbohydrates; TDN, total digestible nutrients.

1) (ADE−/B-blend−) = no vitamin supplementation; (ADE−/B-blend+) = supplementation of a B vitamin blend (thiamine = 28.9 mg/kg MS, niacin = 111.1 mg/kg MS, biotin = 3.3 mg/kg MS); (ADE+/B-blend−) = supplementation with a fat-soluble vitamin blend (A = 6,666.7 UI/kg MS, D = 5,111.1 UI/kg MS (13% D3, 87% Hy-D), E = 70 UI/kg MS); (ADE+/B-blend+) = supplementation with a combination of these two blends.

**Table 3:** Effect of supplementation with different vitamin blends on intake and digestibility of dry matter and diet constituents and microbial production in Nellore finishing bulls

Item	Experimental diets^1)^				p-value		
	
	ADE^(−)^		ADE^(+)^		ADE	B-blend	ADE ×B-blend
	
	B-blend^(−)^	B-blend^(+)^	B-blend^(−)^	B-blend^(+)^			
Number of animals	9	10	9	10	-	-	-
Intake (kg/d)							
DM	6.39_±0.252_^2)^	6.02_±0.239_	6.68_±0.252_	6.11_±0.239_	0.442	0.064	0.701
Ashs	0.28_±0.011_	0.26_±0.010_	0.29_±0.011_	0.27_±0.010_	0.452	0.071	0.702
OM	6.11_±0.241_	5.76_±0.228_	6.39_±0.241_	5.85_±0.228_	0.444	0.063	0.695
CP	0.76_±0.030_	0.71_±0.028_	0.79_±0.030_	0.72_±0.028_	0.447	0.066	0.649
EE	0.19_±0.008_	0.18_±0.007_	0.19_±0.008_	0.18_±0.007_	0.440	0.085	0.701
apNDF	1.43_±0.057_	1.35_±0.054_	1.49_±0.057_	1.37_±0.054_	0.454	0.066	0.683
NFC	3.80_±0.149_	3.58_±0.142_	3.97_±0.149_	3.64_±0.142_	0.441	0.064	0.700
TDN	4.54_±0.183_	4.26_±0.173_	4.87_±0.183_	4.29_±0.173_	0.325	0.022	0.412
N	0.12_±0.005_	0.11_±0.004_	0.12_±0.005_	0.11_±0.004_	0.396	0.097	0.530
Digestibility of nutrients (g/kg)							
DM	680.5_±7.75_^2)^	681.6_±7.36_	701.6_±7.75_	676.8_±7.36_	0.285	0.126	0.096
Ashs	451.6_±29.53_	448.6_±28.01_	480.5_±29.53_	455.6_±28.01_	0.536	0.630	0.706
OM	701.0_±7.57_	700.7_±7.18_	720.4_±7.57_	697.0_±7.18_	0.296	0.118	0.128
CP	632.3_±10.44_	639.1_±9.91_	660.0_±10.44_	642.3_±9.91_	0.138	0.598	0.238
EE	806.5_±11.70_	803.3_±11.10_	802.9_±11.70_	789.8_±11.10_	0.458	0.479	0.665
apNDF	342.3_±19.27_	355.3_±18.28_	358.2_±19.27_	346.6_±18.28_	0.846	0.969	0.517
NFC	850.2_±8.98_	843.6_±8.52_	869.1_±8.98_	839.5_±8.52_	0.401	0.046	0.198
Microbial production							
MCP (g/dia)	522.5_±35.29_^2)^	540.1_±33.48_	573.0_±35.29_	473.2_±33.48_	0.812	0.240	0.096
Efic. (g MCP/kg TDN)^3)^	120.6_±6.54_	130.8_±6.20_	125.0_±6.54_	118.4_±6.20_	0.533	0.787	0.195
Efic. (g MCP/kg DOM)^3)^	126.9_±6.82_	137.5_±6.47_	131.3_±6.82_	124.4_±6.47_	0.515	0.785	0.194

DM, dry matter; OM, organic matter; CP, crude protein; EE, ether extract; apNDF, neutral detergent fiber corrected for residual ash and residual nitrogenous; NFC, non-fiber carbohydrates; TDN, total digestible nutrients; MCP, microbial crude protein; DOM, digestible organic matter.

1) (ADE−/B-blend−) = no vitamin supplementation; (ADE−/B-blend+) = supplementation of a B vitamin blend (Thiamine = 28.9 mg/kg MS, niacin = 111.1 mg/kg MS, biotin = 3.3 mg/kg MS); (ADE+/B-blend−) = supplementation with a fat-soluble vitamin blend (A = 6,666.7 UI/kg MS, D = 5,111.1 UI/kg MS (13% D3, 87% Hy-D), E = 70 UI/kg MS); (ADE+/B-blend+) = supplementation with a combination of these two blends.

2) Values subscripted next to each mean refer to the standard errors of the mean (SEM).

3) Efic (g MCP/kg TDN), microbial efficiency in grams of crude microbial protein per kilogram of TDN; Efic (g MCP/kg DOM), microbial efficiency in grams of crude microbial protein per kilogram of DOM.

**Table 4:** Effect of vitamin supplementation on nitrogen (N) balance in Nellore finishing bulls

Item	Experimental diets^1)^				p-value		
	
	ADE^(−)^		ADE^(+)^		ADE	B-blend	ADE ×B-blend
	
	B-blend^(−)^	B-blend^(+)^	B-blend^(−)^	B-blend^(+)^			
Number of animals	9	10	9	10	-	-	-
N intake (g/d)	119_±4.2_^2)^	112_±4.1_	116_±4.2_	118_±4.1_	0.518	0.632	0.303
N feces (g/d)	43.9_±2.08_	40.6_±1.97_	40.0_±2.08_	42.6_±1.97_	0.869	0.646	0.151
N urine (g/d)	43.7_±2.50_	42.4_±2.37_	46.2_±2.50_	45.4_±2.37_	0.665	0.265	0.926
N absorbed (g/d)	74.9_±2.88_	71.1_±2.73_	76.4_±2.88_	75.4_±2.73_	0.401	0.300	0.624
N retain (g/d)	31.2_±1.40_	28.7_±1.33_	30.2_±1.40_	30.0_±1.33_	0.336	0.887	0.399
Nr:Ni^3)^	26.4_±1.09_	25.7_±1.03_	26.1_±1.09_	25.6_±1.03_	0.587	0.843	0.940

N intake, N consumption; N feces, fecal excretion of N; N urine, urinary N excretion; N absorbed, N absorption; N retained, N retention; Nr:Ni, ratio between consumed and retained nitrogen.

1) (ADE−/B-blend−) = no vitamin supplementation; (ADE−/B-blend+) = supplementation of a B vitamin blend (thiamine = 28.9 mg/kg MS, niacin = 111.1 mg/kg MS, biotin = 3.3 mg/kg MS); (ADE+/B-blend−) = supplementation with a fat-soluble vitamin blend (A = 6,666.7 UI/kg MS, D = 5,111.1 UI/kg MS (13% D3, 87% Hy-D), E = 70 UI/kg MS); (ADE+/B-blend+) = supplementation with a combination of these two blends.

2) Values subscripted next to each mean refer to the standard errors of the mean (SEM).

3) Ratio between retained and consumed nitrogen (value multiplied by 100).

**Table 5:** Effect of vitamin supplementation on animal performance and carcass traits in Nellore finishing bulls

Item	REF^2)^	Experimental diets^1)^				p-value		
	
		ADE(−)		ADE(+)		ADE	B-blend	ADE ×B-blend
	
		B-blend(−)	B-blend(+)	B-blend(−)	B-blend(+)			
Number of animals	5	9	10	9	10	-	-	-
BWi (kg)	-	265_±9.2_^3)^	258_±8.8_	266_±9.2_	257_±8.8_	0.991	0.387	0.894
BWf (kg)	261	445_±13.0_	422_±12.3_	446_±13.0_	427_±12.3_	0.800	0.104	0.875
EBWi (kg)	-	237_±8.3_	231_±7.9_	238_±8.3_	230_±7.9_	0.991	0.387	0.894
EBWf (kg)	234	408_±12.3_	386_±11.7_	410_±12.3_	390_±11.7_	0.811	0.089	0.962
NCW (kg)	89	149_±4.3_	142_±4.1_	151_±4.3_	142_±4.1_	0.808	0.060	0.877
ADG (kg/d)	-	1.25_±0.057_	1.14_±0.054_	1.26_±0.057_	1.19_±0.054_	0.608	0.120	0.693
EBWG (kg/d)	-	1.19_±0.052_	1.08_±0.050_	1.20_±0.052_	1.12_±0.050_	0.624	0.075	0.834
HCG (kg/d)	-	0.77_±0.034_	0.72_±0.032_	0.75_±0.034_	0.74_±0.032_	0.946	0.268	0.594
CCW (kg)	-	258_±8.5_	244_±8.1_	259_±8.5_	247_±8.1_	0.823	0.131	0.882
HCW (kg)	155	268_±8.3_	256_±7.9_	266_±8.3_	258_±7.9_	0.995	0.211	0.824
SFT (mm)	-	7.16_±0.809_	6.58_±0.768_	6.33_±0.809_	6.34_±0.768_	0.502	0.717	0.711
CL (cm)	-	127_±1.8_	126_±1.8_	126_±1.8_	125_±1.8_	0.564	0.523	0.978
LMA (cm^2^)	55.9	75.8_±2.57_	73.2_±2.44_	77.1_±2.57_	75.8_±2.44_	0.441	0.449	0.792
HCY (%)	-	60.3_±0.54_	60.7_±0.52_	59.8_±0.54_	60.4_±0.52_	0.437	0.336	0.842

BWi, initial mean body weight; BWf, final mean body weight; EBWi, initial empty body weight; EBWf, final empty body weight; NCW, weight of non-carcass components; ADG, average daily earning; EBWG, empty body weight gain; HCG, hot carcass gain; CCW, cold carcass weight; HCW, hot carcass weight; SFT, subcutaneous fat thickness; CL, carcass length; LMA, *Longissimus lumborum* muscle area; HCY, hot carcass yield.

1) (ADE−/B-blend−) = no vitamin supplementation; (ADE−/B-blend+) = supplementation of a B vitamin blend (thiamine = 28.9 mg/kg MS, niacin = 111.1 mg/kg MS, biotin = 3.3 mg/kg MS); (ADE+/B-blend−) = supplementation with a fat-soluble vitamin blend (A = 6,666.7 UI/Kg MS, D = 5,111.1 UI/kg MS (13% D3, 87% Hy-D), E = 70 UI/kg MS); (ADE+/B-blend+) = supplementation with a combination of these two blends.

2) REF = reference group, reference animals were used to estimate the initial empty body weight, carcass, non-carcass and body composition of the other experimental animals. They were not included in the statistical analysis.

3) Values subscripted next to each mean refer to the standard errors of the mean (SEM).
